# What is the urban in contemporary Amazon? Implications for health surveillance in the biome

**DOI:** 10.1590/0102-311XEN129723

**Published:** 2023-09-18

**Authors:** Ana Cláudia Duarte Cardoso, Ana Paula dal’Asta, Antonio Miguel Vieira Monteiro

**Affiliations:** 1 Instituto de Tecnologia, Universidade Federal do Pará, Belém, Brasil.; 2 Instituto Nacional de Pesquisas Espaciais, São José dos Campos, Brasil.

Bertha Becker’s provocation ^1^ made in 1995, when referring to the Amazon as an Urbanized Forest, was a milestone. It was the recognition, by an important non-Amazonian and active voice in the debates that characterized the Amazon as a frontier of capitalist accumulation, that the forest was an urban frontier ^2^ ^,^ ^3^. This perspective emerged from the developmentalist narrative for that vast rainforest landscape as a wellspring of exploitable resources (minerals, logging, energy, land), one that obscured the human presence prior to colonization. Recent studies ^4^ add new and important evidence that long before the 16th century the region was vibrant and would have housed at least ten million inhabitants, organized into peoples and territories who mastered soil, water and biodiversity management technologies.

From the native standpoint, there were no large agglomerations that differed from the agricultural productive environment, but rather settlements dispersed along rivers separated by gradients spanning from the inhabited area to the farm, the orchard and the forest, articulated between each other, and with the larger centers, forming networks that made housing and production viable. This paradigm reoriented colonial agriculture, but was disregarded by the urban-industrial paradigm which blindly and in an authoritarian manner reorganized urbanization strategies in Brazilian territories.

Native urbanization, which completely assimilated the forest, and the urban specificities in the Amazon, were not only not understood as such, but were in fact absent from the national agendas of governments, academia (outside the region) and the third sector for most of Brazil’s history. And, as Bertha Becker argued, “*what is not on the agenda, does not exist*”. The production of worrying landscapes and indicators under the climate-environmental agenda and its repercussions on the economic agenda, resulting from the imposition of urbanization patterns according to the urban-industrial logic, revived this debate.

On the other hand, these urbanization processes were intrinsically associated with health-disease processes. Urbanization has altered the distribution of diseases relevant to public health along occupation gradients in transformed landscapes. For public policies associated with health surveillance and control actions, the use of normative definitions of “city” and “countryside” or “urban” and “rural” helped to distinguish between urban and rural health and, consequently, to design actions. Adopting dichotomous categories to explain the differences in risk of exposure as well as disease prevention seemed sufficient. But not for the Amazon.

Over the centuries, despite the brutal reduction of the original population due to diseases and acculturation, the records of a non-binary spatial organization remain strongly manifest in the biome. The legacy of the colonizer’s logic, based strictly in terms of the city-country dipole, and later expressed in the urban-rural dipole, failed to erase such records. More importantly, recognizing the diversity and heterogeneity of their practiced territories ^5^ would be a mobilizing element to establish clear and adequate associations in epidemiological studies and to allocate interventions where needed.

## What then is this urban in contemporary Amazon?

The various socio-territorial typologies present in the region, beyond the cities, and their territorial configurations and reconfigurations, express the historical record of pre-colonial system dynamics that insist on resisting. In the 17th century, the Jesuits’ strategy consisted in setting up directories, which drained the forest production to the reductions located in the village sites, and from there were sent by river to Europe. The Portuguese Crown appropriated this apparatus during the Pombaline administration, transforming those reductions into villages and cities ^6^. In this phase, a hybrid formation was established between indigenous rationality and colonizing logic, which associated with miscegenation fixed previous nomadic families and based the shift from the colonial economy towards a dynamic structured by extractivism, but not only ^7^ ^,^ ^8^. Agriculture and livestock farming were added to riverine dwellings located on the floodplains of the great rivers. Village production fed the city-fairs, which constituted themselves as hubs according to a pattern specific to mercantile economies ^9^.

Descriptions of the region as an empty space by the hegemonic literature appear in the 20th century based on a perceived isolation, or the small size of the existing agglomerations, assumed as dispersed and incompatible with the territorial organization proposed by the regional and urban planning policies and strategies disseminated from the 1940s on. Strategies that relied on an ideological and racist reinforcement (March to the West ^10^), in which riverine, indigenous peoples, peasants, *quilombolas*, who lived in these small agglomerations, were denied the status of civilized. Ideology resumed during the military dictatorship when the narrative shifted into “land for the landless”. The urban-rural dichotomy was thus established, simplifying the balance between society and nature of previous phases, and setting forth enormous challenges due to the incomplete conversion of that native logic by the new agribusiness dynamics and the nascent industrial economy.

The city became a symbolic representation of the urban, complemented by logistical and support structures for new neo-extractive activities (mining, agriculture, livestock, logging economies), which articulated the region’s cities with exogenous urban metabolisms. While the trades linked to the biome economy ^11^ (extraction of non-timber products, production of small boats, food processing, etc.), linked to the territorial scale of villages, hamlets and communities, came to be identified as rural. Productive activities carried out outside the cities consolidated the image of the Amazon as a rural region, demonstrating total incomprehension of a culture based on relations with the forest established over thousands of years. Common sense dictated that the Amazon had no history, lacked its own technological repertoires of value, and thus could be erased quickly. The 20th century ideological political orientation excluded thousands of riverine and forest peoples from access to various public policies, including those health-related.

Incomprehension of the ways of living and producing in the same territory, or of patterns of mobility and daily circulation between cities, villages, hamlets, communities, houses, gardens, orchards, rivers and forest, led to authoritarian planning solutions, disjointed from this reality. These led to an inability to, on the one hand, ensure citizenship by means of recognizing the environmental impact of the new productive activities on those territories by contamination of surface water and soils. And on the other, the inability to recognize endogenous and more appropriate technologies for such key issues as public services and urban infrastructures, such as the supply of drinking water, sanitary sewage and solid waste management ^12^ in the biome, essential to health promotion.

In contemporary Amazon, more strongly than in any other biome, new and diverse forms of settlements and circulation arrangements have come together and function as nodes of a multi-center urban system. They are districts, hamlets, communities, villages, centers of commercial and service operations, logging companies, farms, peasant arrangements, rubber plantations, gold mines, large construction camps, agrarian reform settlements, landless encampments, new quilombos and new indigenous areas. Cities, but mainly their extended urban fabric, using these diversified networks of settlements, linked together by varying degrees of connectivity that define different centralities, conform the new territories of an extensive urbanization ^13^ ^,^ ^14^ ^,^ ^15^ ^,^ ^16^.

This urban extending from the cities mobilizes an extended peri-urban mesh ^17^, made invisible and/or homogenized by the label of rural. A territory that extends between the new and the historic city and the cycles and processes of nature that resist in the socio-spatial typologies located there, but under strong pressure for their disappearance. Hence, this network of centralities configure localities between rivers and forests, establishing an urban-rural continuum rather than an urban-rural dipole. We must shift the agenda from this urban-rural duality. The Amazon never had or has isolated localities; what exists is an intentional invisibility of these socio-spatial processes. This reality increases the complexity of municipalities and the territorial management of their policies, particularly those health-related.

Using a representation based on the urban-rural continuum to characterize the territories realized in one of the main malaria hotspots of the Americas - northwestern Acre - emerged as a better territorial support for developing intervention strategies that could minimize the disease’s burden ^18^. Results showed, regarding malaria, that understanding the place occupied by each locality in its locally referenced urban-rural continuum allowed to develop actions more adequate to their specificities.

Great are the methodological challenges for constructing representations of this extensive urban and extended peri-urban territories. Even greater are the challenges for its operational construction in the context of health services. But such difficulties should be seen as opportunities for new debates on the possibilities of integrating localities into health information systems. We must urgently face the contemporary complexities of this urban reality. In the Amazon, both within and outside the cities, the forest and rivers are means of production and channels of circulation and encounter of all forms of life. And it is in these spaces that old and new health-disease processes (re)emerge.
